# Genetics and genomic medicine in Indonesia

**DOI:** 10.1002/mgg3.284

**Published:** 2017-03-29

**Authors:** Yulia Ariani, Purnomo Soeharso, Damayanti R. Sjarif

**Affiliations:** ^1^ Human Genetic Research ClusterIndonesian Medical Education and Research InstituteJakartaIndonesia; ^2^ Department of Medical BiologyFaculty of MedicineUniversitas IndonesiaJakartaIndonesia; ^3^ Department of PediatricFaculty of MedicineCipto Mangunkusumo National Referal HospitalUniversitas IndonesiaJakartaIndonesia

## Abstract

Genetics and genomic medicine in Indonesia.

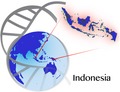

## Introduction

Genetics and genomic medicine are not well recognized in Indonesia due to the lack of awareness about the importance of its future implementation. Only a few centers, at university or hospital based, have already applied a small part of the available methods in doing diagnostic test and studies. Limited facilities are considered as the main issue in accelerating these efforts. The high cost of genetic evaluations and studies have also become an obstacle, not only for patients but for researcher as well. Few numbers of genetic and genomic studies have been published annually from several research centers, yet did not really represent Indonesia because of the vast diversity of its population.

This article aimed to introduce what has been done in Indonesia since the 1970s regarding implementation of genetics and genomic in medicine. Although lack of resources, health professionals and researchers in Indonesia are forced to be able to adapt with the rapid development of science and technology in genetics and genomic, some internal strength, obstacles and opportunities must be addressed and evaluated to optimize this effort.

## Demography and General Statistics

Republic of Indonesia is an archipelago located in Southeast Asia, which consists of five main islands and more than 13,000 small islands. Having land area of 1,922,570 square kilometers and water territory of 3,257,483 square kilometers, Indonesia becomes the largest archipelago in the world. This huge archipelagic country is extending 5120 km from east to west and 1760 km from north to south. Indonesia has a strategic position because it lies between two continents (Asia – Australia) and two oceans (Pacific ocean – Indian ocean) (Geospatial Information Bureau, 2017).

**Figure 1:**
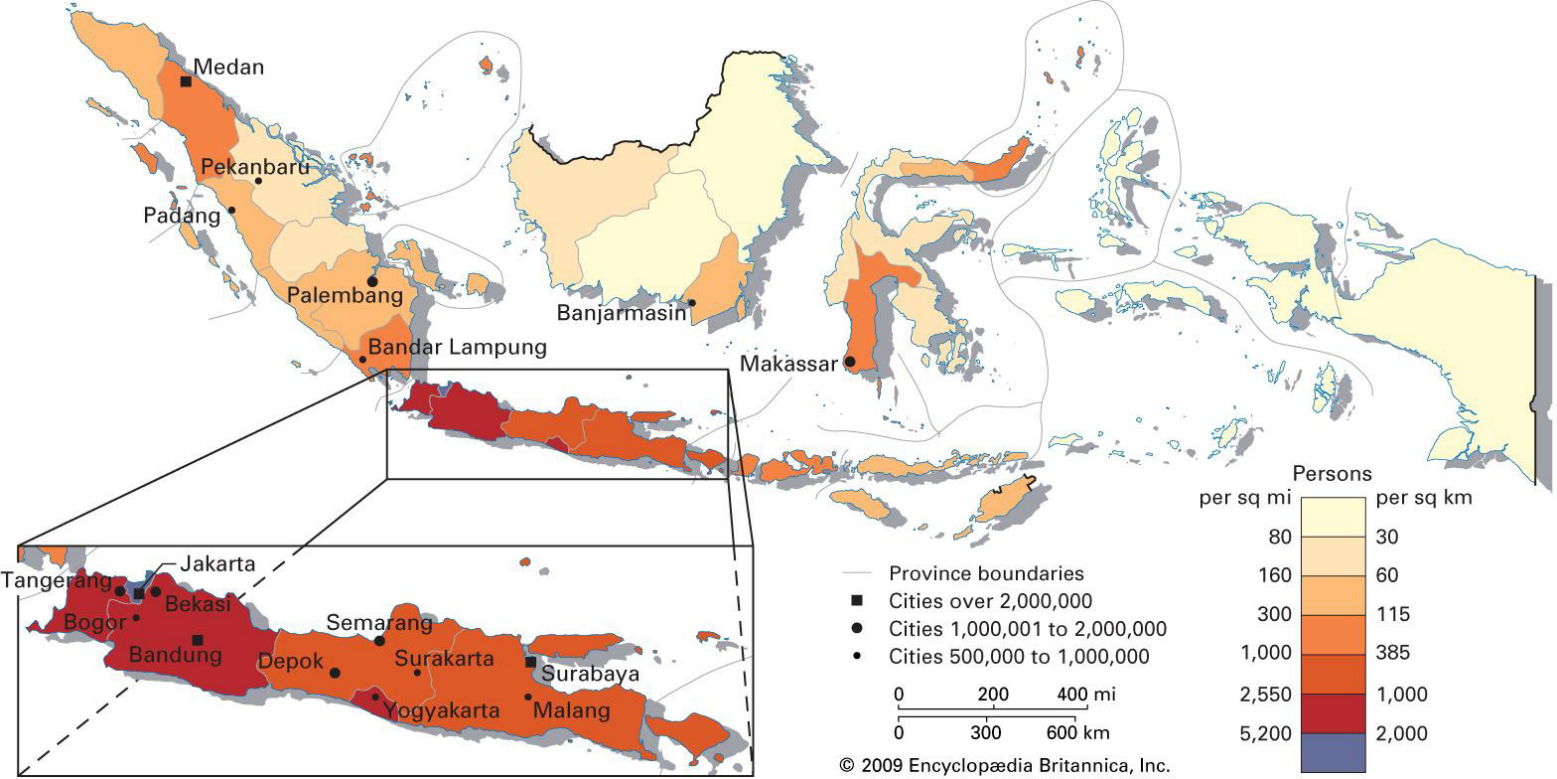
Map of Indonesia and its population density. (Encyclopedia Britannica, 2017).

Estimated population number in 2015 is 255,461,686 with mean population density of 133,5 inhabitants per square kilometers. Figure 1 gives illustration of Indonesia archipelago and its population which shows an uneven distribution. Java island (red color) is the most dense island, with mean density of over than 250 inhabitants per square kilometers. Jakarta, the capital of Indonesia, is the most dense city in Java island, with density of 15,327 inhabitants per square kilometer. About 66.6% of Indonesian population is urban (Data and information center MoH RI, 2015; Indonesian Center of Statistic Bureau 2015; Encyclopedia Britannica, 2017).

Total Growth Domestic Product (GDP) stood at 11,540.8 trillion IDR in 2015 or 45.2 million IDR per capita. Economic growth still went down since 2012 due to global economic crisis, and it reached the number of 5.04% in 2015 compared to 6.03% in 2012. Half of the income per capita was spent for food (50.04%). Three major expenses are housing/household needs (20.75%), foods/drinks (13.37%), and goods/services (12.35%). Family health expense is only as small as 3.29% of the total expenses (Data and information center MoH RI 2015; Indonesian Center of Statistic Bureau 2015).

Only 49.23% population finished their elementary and intermediate education in 2015. Although Indonesia government has already implemented free education for elementary and intermediate school, the lack of facilities is still a big issue to be addressed. Illiterate number has decreased from 7.56% in 2011 to 4.78% in 2015 (Indonesian Center of Statistic Bureau 2015).

Most of Indonesian people are Muslims (87.18%). The rest consists of Christians (6.96%), Catholics (2.9%), Hindus (1.69%), Buddhists (0.72%), Confucians (0.05%), and others (0.13%) (Indonesian Center of Statistic Bureau 2010). There is only little information indicating consanguinity in Indonesia. Nevertheless, current data indicate that 10.4% of the 6.7 billion global population are related as second cousins or closer (*F* ≥ 0.0156) (Bittles 2008).

## Health Indicators and Health Services

Maternal mortality rate and infant mortality rate is still considered as a significant health burden for Indonesia. Although efforts have been done to address these issues, the number is still high. Maternal mortality rate remained high over the last decade (228 per 100,000 life birth), whereas infant mortality rate only decreased at the beginning of the establishment of Millenium Development Goal (MDG) 5, and remained steady after 2012. Despite the successful management of infectious diseases in neonates and infants, the mortality rate could not significantly be decreased (UNICEF Indonesia, 2012).

There is a slight increase in life expectancies for men and women, 67.9 year old in 2010; 68.9 year old in 2014 and 71.8 year old in 2010; 72.6 year old in 2014, respectively. Percentage of delivery by health professionals has been increased since 2010 until 2015, 79.82% and 91.51%, respectively. But there is a decrease in immunization coverage of under five‐year children. After the initiation of Social Safety Net Program for Health in 1998, there is a significant decrease in using traditional/alternative health facilities, and increase in formal health facilities visits (Indonesian Center of Statistic Bureau 2015).

Having a huge territory, Indonesian government faces serious challenge to fulfill the need of proper health facilities. Primary health centers (PHCs) became the spearhead of national health service system. There was a slight increment in number of PHCs from 2011 until 2015 (9321 and 9754 respectively). But the ratio of PHCs number per 30,000 population was actually decreased (Data and information center MoH RI, 2015).

At early 2016, Indonesian government initiated the national health insurance program called “Badan Penyelenggara Jaminan Sosial” (BPJS). This program allowed the expansion and equitable distribution of health care coverage. There were still limitations in the type of health services, however with a good communication and advocacy between health providers and government there will always be opportunity for negotiations, regarding the optimal health services for community (Ministry of Health Republic of Indonesia 2016a,b).

## Genetic Diseases in Indonesia

Genetic diseases have not been considered as a serious condition in Indonesia. The high incidence of infectious diseases has drained the human resources and health costs. Compared with any other developing countries in Southeast Asia, such as Malaysia, Thailand, Philippines, and Vietnam, Indonesia seems to be left behind. There is lack of data about prevalence of genetic diseases. Surveillance for genetic conditions has not been done systematically. Research in genetic diseases is very few because of limited source of fund and access to advance technology as well. Genetic diseases were considered as an incurable condition, therefore not many researchers are interested in this field.

Despite the success of reducing the number of premature births and infectious diseases in neonates, Indonesia is still facing a high neonate and infant mortality rate. Birth defects emerge as a significant contributor toward this mortality rate. Birth defect accounts for 1.4% to neonatal deaths in 2007, and this number increase as high as 10.5% in 2010. (National Basic Health Research 2007 Ministry of Health Republic of Indonesia 2008; Child Health Epidemiology Reference Group World Health Organization 2010). Congenital malformation, as one of the cause of birth defects, contributes about 5.7% of infant mortality and 4.9% of under‐five mortality. This number increased as high as 19% toward infant mortality in 2010 (Ministry of Health Republic of Indonesia 2010).

Congenital malformation is considered to be the most important genetic condition worldwide, because it can cause early mortality and significant health burden. More than 90% neonates born with congenital malformation are from low‐middle income country, which have limited source of health cost. (Christianson et al. 2006). There are limited national data about congenital malformation in Indonesia. A five‐year observation among infants born in Manado, Indonesia, reported the incidence of congenital malformation was 0.9%. The most common malformations were cleft lip and palate, talipes, anal atresia, omphalocele and congenital heart diseases. (Masloman et al. 1991).

Data from Medical Biology Department Faculty of Medicine Universitas Indonesia (FMUI) reported 103 congenital malformation patients, from January 2011 until June 2013, sent to cytogenetic laboratory to have chromosome examination. Table 1 describes clinical diagnosis of these patients.

**Table 1:** Clinical diagnosis of patients sent to cytogenetic laboratory FMUI (Ariani et al. 2014)

Type of congenital malformation and karyotyping results	Number	Percentage (%)
Known syndrome (*n* = 67)		
Down syndrome	55	78.6
Turner syndrome	9	12.9
Klinefelter syndrome	1	1.4
Edward syndrome	2	2.9
Unknown syndrome (*n* = 36)		
46,XY,add(13)(q34)	1	0.03
46,XY,6 Mar, 17 dmin	1	0.03
46,XX,r(4)(p16q35)	1	0.03
46,XY,22ps+	1	0.03
46,XY,add(5)(p15)	1	0.03
47,XX+G	1	0.03
46,XX/45XX Rob (13,15/q10.2,q10), 45XX Rob (13,14)(q10,q10)	1	0.03
46,XX, ring 13	1	0.03
Normal karyotyping	29	0.8

Neural tube defects (NTDs) were quite prevalent in Jakarta, although the number was low compared to Beijing and Kuala Lumpur. Respectively, predicted NTD rates were highest at Beijing (30 per 10,000 life birth), followed by Kuala Lumpur (24 per 10,000 life birth), and lowest in jakarta (15 per 10,000 life birth) (Green et al. 2007). Fronto‐ethmoidal encephalomeningocele, is a rare type of NTD, occur about 1 per 5.000 population in Asian countries, including Indonesia (Bhattacharjee et al. 2008).

Thalassemia is the major genetic problem in many Asian countries, including Indonesia. As a genetic disorder, thalassemia has its specific characteristic due to the wide variability of the mutations. The diversity of the genetic background can be seen in the carrier frequency of *β*‐thalassemia (5–10%), Hb E (1–33%) and ɑ‐thalassemia (6–16%) from the various ethnic population. This variation resulted to unequal anticipated carrier testing and prenatal testing workload. Therefore, carrier screening protocol and prenatal testing have to be designed on a regional basis (Wahidiyat et al. 2006).

Down syndrome is a common genetic condition found in Indonesia. Data from the Down Syndrome Association of Indonesia, stated that almost 300,000 cases of Down syndrome in the country. According to the Indonesia Health Profile the prevalence of Down syndrome is 0.12% (Ministry of Health Republic of Indonesia 2010). Cytogenetic laboratory of Medical Biology Department FMUI reported 32 cases confirmed of Down syndrome in 2015 and 19 cases in 2016. There are three other main cytogenetic laboratories in the country that may have the same number of testing for Down syndrome confirmation (Ariani et al. 2014).

Like in many other Southeast Asian countries, G6PD deficiency is the most common enzyme deficiency disorder in Indonesia. Global prevalence is reported about 4.9%. Its incidence can be as high as 25% in some populations (Nkhoma et al. 2009). Report from Cipto Mangunkusumo National Referral hospital, G6PD deficiency affects approximately 2.66% of full‐term babies. (Suradi et al. 1979). Whereas, among neonatal hyperbilirubinemia the proportion of G6PD deficiency was 19.3% (Wahidiyat 1996). A study in Thailand showed the prevalence of G6PD Canton (1376G>T) was 10% and G6PD Kaiping (1388G>A) was 5% of G6PD‐deficient neonates with hyperbilirubinemia (Nuchprayoon et al. 2002). In Malaysian Chinese, the prevalence of G6PD Canton was 54.3% and G6PD Kaiping was 37.1% among hyperbilirubinemia neonates (Ainoon et al. 1999).

Recent study from a private Woman and Children Hospital at Jakarta reported the prevalence of G6PD deficiency was 6.26% for male infants and 4.07% for female infants. There was no significant difference in the risk for severe hyperbilirubinemia between G6PD deficient infants compared with normal infants (Kaban and Wijaya 2011).

Degenerative diseases as part of the noncommunicable diseases (NCDs) become an emerging burden toward Indonesian health status for last decade. Despite its high level of morbidity, it also affluent urban people and rural as well. Hypertension is the most common NCDs in Indonesia, with total prevalence of about 31.7% (Indonesia Basic Health Research, 2007). Other NCDs related to genetic backgroud are stroke (cerebrovascular disease) 8.3%, heart problems 7.2%, cancers and tumors 4.3%, asthma 3.5%, and diabetes mellitus 1.1% (Ministry of Health Republic of Indonesia 2010). Stroke is the most common cause of death, it accounts for 15.4%. Other NCDs which also causing deaths are hypertension (6.8%), ischemic heart disease (5.1%) and other heart disease (4.6%) (Ministry of Health Republic of Indonesia 2008). NCDs are known to have multifactorial etiology. Genomic factor, especially epigenomic, is believed to have significant role in the development of these conditions.

## Genetic Services in Indonesia

Genetic service has been started since 1970s invery limited facilities. Medical Biology Department of FMUI provides chromosome examination using Giemsa solid staining which mostly detects number of abnormalities. A brief genetic counseling was given by medical doctors, according to the results of the test. Giemsa banding technique just begun after 2010. Using this method, structural chromosomal abnormality can be detected. Genetic counseling was also given more in‐depth, as a consequence of the findings. Table 2 shows a total of 227 patients sent to this laboratory from January 2011 until June 2013.

**Table 2:** Patient's characteristic according to some variables (Ariani et al. 2014)

Variable	Number	Percentage (%)
Total number of patients 2011–2013 (*n*)	*n* = 227	
Number of patients per year		
January 2011–December 2011	55	24.2
January 2012–December 2012	97	42.7
January 2013–June 2013	75	33.1
Patients distribution according to age group	*n* = 218	
Neonate (0–28 days)	6	2.6
Infant (>28 days–12 months)	64	28.2
Under‐five (>1 y.o–<5 y.o)	56	24.7
School age (>5 y.o–12 y.o)	36	15.9
Adolescent (>12 y.o–18 y.o)	21	9.3
Adult (>18 y.o)	35	15.3
Patients distribution according to sex group	*n* = 222	
Male	81	35.7
Female	99	43.6
Ambiguous	42	18.5
Patients distribution according to clinical diagnosis	*n* = 227	
Un known syndrome	36	15.9
Confirmation of known syndrome	70	30.8
Disorders of sexual development	57	25.1
Malignancy (chronic leukemia)	11	4.8
Infertility	7	3.1
Habitual abortion	5	2.2
Failure to thrive	7	3.1
Amenorrhea	7	3.1
No clinical diagnosis	27	11.9

Molecular cytogenetic analysis has been done since 2014 on research based. Seventy two multiple congenital malformation cases were recruited from July 2013 until June 2014. History taking, physical examinations and imaging studies were done. All findings were compared to phenotype database Online Mendelian Inheritance in Man (OMIM) and Pictures of Standard Syndromes and Undiagnosed Malformation (POSSUM) databases. Based on these, subjects were grouped as “can be diagnosed” or “cannot be diagnosed” (Sjarif and Ariani 2015).

Fifty one subjects can be clinically diagnosed as chromosomal syndromes. Chromosome analysis was performed to confirm the diagnosis. Twenty one of them were considered as an unknown syndrome. Chromosome analysis was also done as a first step of diagnosis strategy. Nine cases among those subjects found to have chromosome aberrations, whereas twelve cases showed normal karyotypes. Type of chromosome aberrations can be seen in Table 1. Only eight out of nine subjects from the normal karyotype group proceed to the microarray step (Sjarif and Ariani 2015).

Microarray examinations were done at Medical Genetics Department, Universitair Medisch Centrum Utrecht, Netherlands, using Infinium CytoSNP‐850K DNA analysis bead chip kit from Illumina. Chips were scanned using Hi‐scan scanner from Illumina. Data were extracted using genome studio software. Data were analyzed using Nexus software. Five out of eight subjects which tested by microarray showed normal array. Two subjects showed well known deletion syndromes, which are Wolf‐Hirschhorn syndrome and Williams‐Beuren syndrome. One case has normal array with two large regions lost of Heterozygosity (Ariani and Sjarif 2015; Sjarif and Ariani 2015).

Eijkman Institute is one of the leading research centers in Indonesia which also developed genetic laboratory. Genetic Counseling Unit is the collaboration of this institute with Department of Obstetrics and Gynecology, Dr Cipto Mangunkusumo Hospital Jakarta, since 1999. This service initiated mainly for thalassemia patients and their family. It became expanded for other diseases such as Spinal Muscular Atrophy (SMA), Duchenne Muscular Dystrophy (DMD), achondroplasia, Down Syndrome, Klinefelter syndrome, Turner syndrome, Disorder of Sexual Development (DSD) cluster, Prader‐Willi syndrome and/or Angelman syndrome. Premarital and preconception counseling was also provided as part of this services. Prenatal diagnosis is done only for aneuploidy detection, using amniocytes sample. DNA forensic unit at The Eijkman Institute gives services such as parentage (paternity and/or maternity) test, Disaster Victim Identification (DVI), and perpetrator identification (Eijkman Institute, 2016).

CEBIOR (Center for Biomedical Research) at faculty of Medicine, Diponegoro University, is also one of the leading centers providing genetic laboratory service in Indonesia since 1999. Genetic testing is mainly for cytogenetic and some of common genetic diseases caused by certain gene mutations. Cytogenetic examination is done from blood, bone marrow, villi chorialis and amniotic fluid. G‐banding is the most frequent method being used, whereas C and silver banding will be done only for certain conditions to identify Y chromosome or other small group of chromosomes. Other genetic testing are being done based on molecular study, such as FMR‐1 gene mutation analysis for Fragile X mental retardation, SRY and AZF gene microdeletion for infertility and genital defect cases, also the Cyp21 gene mutation analysis for Congenital Adrenal Hyperplasia. Some gene mutations are being done on research based, such as polymorphism of PAI‐1 gene, TNF ɑ, Cyp 9, and other cytokine genes (Center for Biomedical Research, 2016).

## Research in Genetics and Genomic Medicine

Research in genetics and genomic medicine has been done sporadically in several research centers in Indonesia. There are five focus topics approved by government as main subjects to be funded, and genomic is one of them. Genomic research is believed that it could be able to accelerate the development of science and technology in Indonesia. Cancer study was developed at Cancer Center of Dharmais Hospital and Hematology‐Oncology Department Dr. Cipto Mangunkusumo National Referral Hospital. Both the hospitals are in Jakarta. Reproductive genetics study is mainly done at Department of obstetrics and gynecology, Dr. Cipto Mangunkusumo National Referral Hospital, Jakarta. Human genomic study related to dengue virus and *Mycobacterium tuberculosis* is one of the research excellence at Microbiology Department FMUI.

Congenital malformation laboratory diagnostic confirmation is mainly studied at Medical Biology Department FMUI, Eijkman Institute and CEBIOR Faculty of Medicine Diponegoro University. Research was basically done to develop methods as diagnostic tools and discover genes which are responsible in certain malformation. Some studies on common diseases also have been done, such as the role of some genes in pathogenesis and management of G6PD deficiency, obesity, atopic dermatitis and wound healing.

Although G6PD deficiency is the most common hereditary disorder in human, Indonesia has very limited data about this disease, especially its genetic data. G6PD deficiency might lead to a serious and fatal condition known as kernicterus. The high level of total serum bilirubin (above 20 mg/dL) was the predictor of developing this condition (Meredith and Beth 2002). Newborn screening for this condition has been applied in many countries as a standard screening for newborn (Bhutani et al. 2010). The cost effectiveness was the main issue in starting this program in Indonesia. Prioritizing which disease has to be screened is very important. For G6PD deficiency condition, genetic mutations data of related gene might help in deciding whether the newborn sreening for this disease has to be prioritized. Unfortunately genetic mutations of Indonesian G6PD deficient infants in Jakarta are still not documented until the year 2005.

A cross‐sectional study conducted in 2006 at Cipto Mangunkusumo National Referral Hospital Jakarta, investigated 36 full‐term G6PD deficient neonates with hyperbilirubinemia. Among these subjects, 11 neonates required blood exchange transfusion (BET) therapy. Diagnosis of hyperbilirubinemia was established by total bilirubin level in the serum more than 10 mg/dL. The BET therapy was performed when total serum bilirubin level was above 20 mg/dL (Lo et al. 1994). Using simple PCR‐based technique with artificial created amplified restriction sites method reference followed by restriction enzyme digest, this study found 1 G6PD Canton variant (1376G>T) and 1 Kaiping variant (1388G>A). Infant with G6PD Canton (1376G>T) has total bilirubin of 32.3 mg/dL and required BET therapy. This infant is Chinese and other etiologies of jaundice had been excluded. Infant with G6PD Kaiping (1388G>A) has a total bilirubin level of 18 mg/dL. This infant is Javanese and other etiologies of jaundice had been excluded as well (Yuniar et al. 2006). This study suggested that in Indonesian population the pathologic mutations related to serious complication of hyperbilirubinemia (kernicterus) was very rare. Thus, newborn screening for G6PD deficiency in Indonesia might not be as cost‐effective as in other countries.

A study of UCP (uncoupling protein) genes polymorphism role in obesity pathogenesis was conducted in 2011–2012, at Semarang, Central Java. Seventy‐six school‐age children (36 obese and 40 healthy, mean age 12.8 year old) was recruited. This study analyzed the differences in energy expenditure among subjects with different type of single nucleotide polymorphism (SNP); UCP3‐55C/T, UCP3 Y210Y, and UCP2 A55V. Total energy expenditure (TEE) of the subjects with the T/T genotype at UCP3‐55C/T after adjusting for fat‐free mass (63.2 ± 7.2 kcal/kg/day) and T/T at UCP2 A55V (62.8 ± 5.6 kcal/kg/day) was lower than that of the subjects with the C/C and C/T genotypes (*P* < 0.05). Resting energy expenditure (REE) of the subjects with these T/T genotypes tended to be lower than that of the subjects with C/C and C/T (*P* ≥ 0.05). No significant differences in REE or TEE were found between the UCP3 Y210Y genotypes (Mexitalia et al. 2013).

The role of apolipoprotein E polymorphism in improving dyslipidemia in obese adolescents following physical exercise and National Cholesterol Education Program (NCEP) step II diet intervention was studied in 2013. This study recruited 60 dyslipidemic obese adolescents which received physical exercise and the NCEP step II diet for 28 days. Apolipoprotein (apo E) genotypes and blood lipid levels were compared before and after interventions. This study concluded that Apo E alleles might influence improvement in lipid profiles after diet and exercise interventions. These results could benefit to the personalized dyslipidemia management in obese adolescent, to determine which person will benefit to be treated with blood lipid drugs (Gultom et al. 2014).

A study of *FLG* gene mutation and *FADS1* and *FADS2* genes polymorphism in atopic dermatitis (AD) patients was done in 2014–2015. Although this study did not find Filaggrin mutation that was reported as pathogenic from NCBI, the frequency of FADS1 polymorphism was 22–27%, whereas FADS2 polymorphism was 15–48%. Strong correlation was seen between genetic variations of FADS genes with the alteration of LCPUFA. Arachidonic acid as the product of LCPUFA was higher in the minor allele compared with the major allele. No association was found between genetic variation of FADS genes and the increased ratio of AA/DHA with the occurrence of AD. Exclusive breast feeding for 3–6 months seems to give protective effect (Tanjung et al. 2016).

Meilany et al., studied the role of GSTP1 gene polymorphism as a risk factor of wound dehiscence in hipoxic state. GSTP1 105V polymorphism did not significantly associate with the level of oxidative stress marker. However, post operative TcPO2 measurement was significantly decreased in patients with Ile/Val genotype. Furthermore, Ile/Val and Val/Val genotypes increased the risk of wound dehiscence in patients who have surgical complications (anemia, hypoalbunemia and septicemia) (Meilany et al. 2016).

Eijkman Institute is focusing its studies on mitochondria and red blood cell disorders. Some on‐going studies are mutation spectrum of alpha and beta thalassemia in patients and population, thalassemia pre‐natal diagnosis: cases and problems, G6PD deficiency in Indonesian population, fetal programming of impaired nutrition: role of malaria infection in pregnancy, maternal genetic factors and response to supplementation during pregnancy, and disorder of sexual development (Eijkman Institute, 2016).

## Conclusion

After more than four decades of working, genetics and genomic medicine still faces a considerable challenge to be addressed. Lack of awareness of health professionals and government, lack of interest of researcher on genetic diseases, limited research funding, limited access to high technology, low national health budget and low income family are seem to be the main obstacles to be overcome in implementation of genetics and genomic medicine. Despite these conditions, several research centers still managed to do some studies and few numbers of genetic testing. Several collaborations with countries abroad have been done to overcome some obstacles. Yet, Indonesia still has to accelerate this effort to be able to catch up its lag. Mentoring and collaborations are needed to enable Indonesia in doing so.
